# Investigating the Basis Set Convergence of Diagrammatically Decomposed Coupled-Cluster Correlation Energy Contributions for the Uniform Electron Gas

**DOI:** 10.1021/acs.jctc.4c00224

**Published:** 2024-07-08

**Authors:** Nikolaos Masios, Felix Hummel, Andreas Grüneis, Andreas Irmler

**Affiliations:** Institute for Theoretical Physics, TU Wien, Wiedner Hauptstraße 8-10/136, 1040 Vienna, Austria

## Abstract

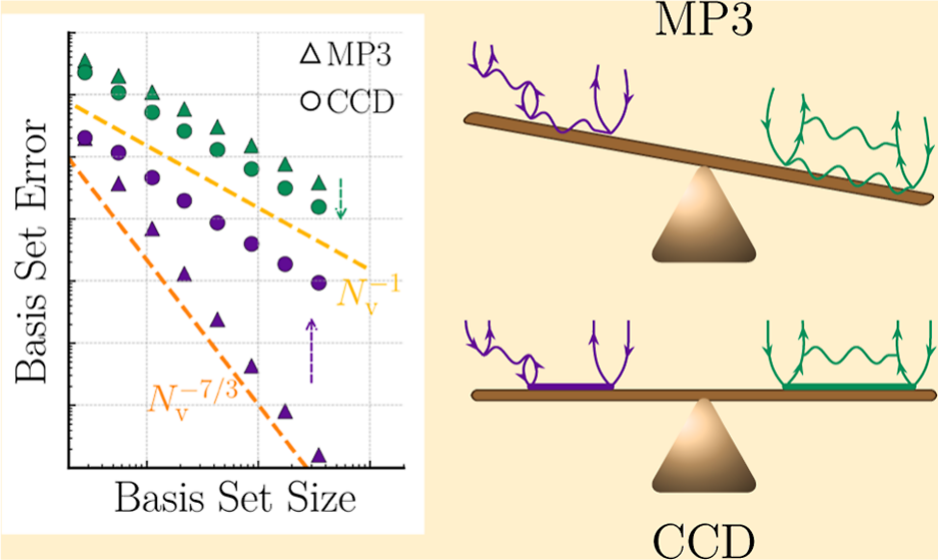

We investigate the convergence of coupled-cluster (CC) correlation energies and related quantities with respect to the employed basis set size for the uniform electron gas (UEG) to gain a better understanding of the basis set incompleteness error (BSIE). To this end, coupled-cluster doubles (CCD) theory is applied to the three-dimensional UEG for a range of densities, basis set sizes, and electron numbers. We present a detailed analysis of individual diagrammatically decomposed contributions to the amplitudes at the level of CCD theory. In particular, we show that only two terms from the amplitude equations contribute to the asymptotic large-momentum behavior of the transition structure factor, corresponding to the cusp region at short interelectronic distances. However, due to the coupling present in the amplitude equations, all decomposed correlation energy contributions show the same asymptotic convergence behavior to the complete basis set limit. These findings provide an additional rationale for the success of a recently proposed correction to the BSIE of CC theory. Lastly, we examine the BSIE in the CCD plus perturbative triples [CCD(T)] method, as well as in the newly proposed CCD plus complete perturbative triples [CCD(cT)] method.

## 1 Introduction

Coupled-cluster (CC) theories are widely used in molecular quantum chemistry and have become increasingly popular for studying solid state systems. CC theories approximate the true many-electron wave function in a systematically improvable manner by employing an exponential ansatz with a series of higher order particle-hole excitation operators. While systems exhibiting strong electronic correlation effects require high-order excitation operators, systems with strong single-reference character can be well described using low-order excitation operators.^1^ In particular, at the truncation level of single, double, and perturbative triple particle-hole excitation operators, CCSD(T) theory predicts atomization and reaction energies for a large number of molecules with an accuracy of approximately 1 kcal/mol.^1^ Although the computational cost of CCSD(T) theory is significantly larger than that of the more widely used approximate density functional theory calculations, recent methodological developments enable the study of relatively complex systems, for instance, molecules adsorbed on surfaces.^2−10^ However, high accuracy compared to experiment can only be achieved if the ansatz is fully converged with respect to all computational parameters that model the true physical system. These include the number of atoms used to model an infinitely large periodic crystal and the truncation parameter of the basis set. Any truncation of the employed one-electron basis set introduces a basis set incompleteness error (BSIE) in CC and related theories.

This work aims at a detailed investigation of BSIE in CCSD and CCSD(T) methods using a plane wave basis set. For a better understanding of the corresponding BSIE we employ the uniform electron gas (UEG) model Hamiltonian, which includes a kinetic energy operator, an electronic Coulomb interaction, and a constant background potential to preserve charge neutrality. The UEG model depends only on parameters that have a well-defined physical interpretation: (i) the electronic density, (ii) the number of electrons and cell shape, and (iii) a momentum cutoff defining the employed basis set. The electronic density controls the relative importance of the kinetic energy operator compared to the Coulomb interaction. In this manner, one can continuously transform the system from a weakly correlated system at high densities to a strongly correlated system at low densities. The number of electrons and the cell shape used to model the electron gas at a fixed density allow for the investigation of finite size effects.^11,12^ Due to the translational symmetry of the UEG model Hamiltonian, the mean-field orbital solutions correspond to plane waves characterized by a wave vector. The momentum cutoff makes it possible to truncate the employed plane wave basis in a systematic manner, making the UEG ideally suited to study BSIEs of electronic structure theories.^13−15^

In this work, we are mainly interested in the BSIEs introduced by large cutoff momenta compared to the Fermi momentum of the UEG. Although the Fermi sphere defines a complete plane wave basis set needed for the representation of the mean-field ground state wave function, the representation of correlated wave functions requires a significantly larger basis set. In particular, at the point where two electrons coalescence, the exact correlated wave function exhibits a cusp.^16−18^ As a consequence, a large number of one-electron orbitals are needed to describe this feature with sufficient accuracy. Apart from increasing the one-electron basis set, it is also possible to account for the cusp in the wave function and derived properties directly by adding basis functions explicitly depending on two electronic coordinates. A variety of techniques have been developed to accelerate the slow convergence to the complete basis set (CBS) limit including explicitly correlated methods,^19−23^ transcorrelated methods,^24−28^ or basis-set extrapolation techniques.^29^ These methods are mainly used for molecular calculations.

Recently, we proposed and investigated a finite basis set correction for the CCSD method that is based on a diagrammatic decomposition of the correlation energy.^30^ We have shown that the finite basis set error is dominated by two contributions to the CCSD correlation energies, corresponding to the second-order energy and a term that we referred to as the particle–particle ladder (PPL) term.^31^ In ref (30) we examined the accuracy of different approximate corrections to the BSIE in the PPL term. Here, we present a more detailed study of the various correlation energy contributions that can be obtained from the diagrammatic decomposition of the CCSD correlation energy. We also investigate other related quantities as functions of the basis set size, the electron number, and the electronic density. This investigation allows us to determine the next-to-leading-order contributions to the basis set error in the UEG.

Our diagrammatic decomposition approach is partly motivated by prominent examples that sum up particular contributions in the perturbation series to infinite order. Famous examples are the resummation of ring-type contributions, as demonstrated in the random phase approximation (RPA),^32^ and ladder theory (LT),^33,34^ which contains PPL contributions. CCSD contains all diagrams appearing in RPA and LT, as well as many further contributions beyond that. This feature alone makes CCSD interesting, as both RPA and LT are known to have prominent failures. The RPA lacks an accurate description of the short-range electronic correlation. Already at medium densities the pair-correlation function gets negative for vanishing interelectronic distances.^35^ On the other hand, the RPA is known for providing an accurate description in the long-range regime.^36^ In contrast, short-range electron correlation can be properly described by the LT.^37^ Apart from that, in the particular case of systems with long ranged Coulomb interactions, LT was found to be unsatisfactory.^38^

We present the theoretical framework in Section 2 and describe the computational details in Section 3. Section 4 is devoted to the BSIE of the coupled cluster doubles (CCD) method and related theories, whereas Section 5 discusses the BSIE for the studied methods, including triple excitations.

## 2 Theory

### 2.1 Uniform Electron Gas

In this work, we study the UEG model system with *N* electrons within a finite simulation cell under periodic boundary conditions. A positive background charge ensures charge neutrality in the unit cell. We work with spatial orbitals, each occupied by two electrons with opposite spin. We will restrict the analysis to cubic boxes, with a box volume Ω defined by the electron number *N* and the density parameter *r*_s_. The latter defines the radius of a sphere whose volume is equal to Ω/*N*.^39^ Throughout this work, we employed Hartree atomic units.

**Figure 1:**
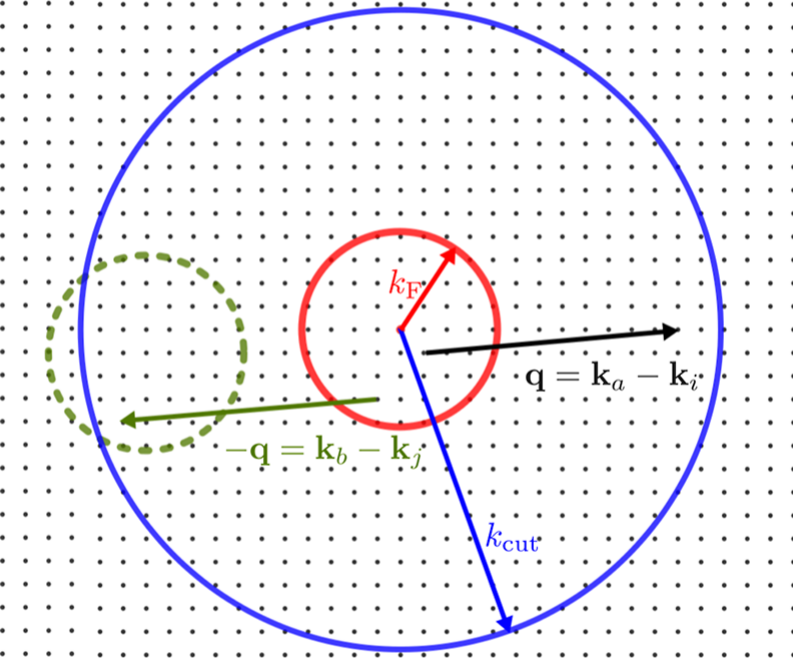
Illustration of the reciprocal grid: Wave vectors of occupied orbitals are found inside the Fermi sphere of radius *k*_F_, shown as a red circle. The wave vectors of virtual states in the finite basis are located outside the Fermi sphere but inside the cutoff sphere of radius *k*_cut_, shown as a blue circle. The wave vectors of the remaining infinite augmented virtual states are located beyond the blue sphere. The black arrow indicates a one-body excitation process with momentum transfer ***q*** from an occupied orbital ***k***_*i*_ to a virtual orbital ***k***_*a*_. The green circle depicts the set of all non-vanishing ***k***_*b*_ in the four-index Coulomb integral υ_*ij*_^*ab*^ for two given states *i* and *a* with the momentum transfer ***q*** = ***k***_*a*_ – ***k***_*i*_. Note that depending on the momentum transfer ***q***, the set of non-vanishing virtual states can be located either entirely inside, entirely outside, or partially outside the radius *k*_cut_.

For the UEG, plane waves are solutions of the Hartree–Fock (HF) Hamiltonian
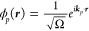
1where the wave vectors ***k***_*p*_ represent reciprocal lattice vectors of the simulation cell. The corresponding HF eigenenergies are
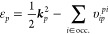
2where *i* labels occupied orbitals. The two-electron Coulomb integrals are given by
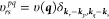
3with the momentum transfer vector ***q*** = ***k***_*p*_ – ***k***_*r*_ and the corresponding Coulomb interaction  for ***q*** ≠ **0**. At ***q*** = **0** the Coulomb interaction is singular. However, this singularity is integrable and various techniques exist to resolve it (see ref (40) and references therein). In this work we employ the regularization as described by Fraser et al.,^41^ which is interpreted as the potential at the unit point charge due to its background of its own periodic images. Oftentimes this term is also referred to as the Madelung term. An important characteristic of the UEG is the conservation of momentum. As evident from eq 3, Coulomb integrals are non-zero only if the momentum transfer vector of the left indices is identical to the negative transfer vector of the right indices (see Figure 1). For sufficiently large densities, as employed in this work, the HF orbital energies are strictly ordered by the length of the corresponding wave vector.

Here and throughout this article we use the following index labels

- *i*, *j*, *k*, ... occupied states,
- *a*, *b*, *c*, ... virtual states in the finite basis set,
- α, β, γ, ... virtual states beyond the finite basis set, referred to as *augmented virtual states*.

*p*, *q*, *r,* and *s* are used to label states that may be either occupied or virtual.

We restrict ourselves to the paramagnetic UEG. Therefore, the number of occupied states *N*_o_ is half the number of electrons *N*. The momentum of the occupied orbital with the highest eigenenergy is called the Fermi wave vector, *k*_F_, and the sphere of radius *k*_F_ is called the Fermi sphere.

The number of virtual states *N*_v_ is determined by a plane-wave cutoff momentum *k*_cut_ which is typically much larger than *k*_F_. The number of virtual states *N*_v_, contained between the spheres with radius *k*_cut_ and *k*_F_ is proportional to *k*_cut_^3^.^13^ We stress that in the UEG the orbitals are unchanged if the number of virtual orbitals is changed (cf. the generalized Brillouin condition in explicit correlated methods^21^).

We refer to the infinite number of plane waves with momentum exceeding *k*_cut_ in magnitude as *augmented virtual states*. These states are considered theoretically only to better understand and investigate BSIEs of central quantities and various contributions to the correlation energy. Consequently, there appear contributions, for instance, Coulomb integrals, involving one state from the virtual states in the finite basis and another state from the augmented basis set, viz. υ_*ij*_^aβ^. For a given choice of *a* and *i*, momentum conservation dictates that the number of non-zero choices for β and consequently *j* is bounded by the number of occupied states *N*_o_. This is illustrated in Figure 1. Thus, these contributions will be negligible in the limit of an infinitely large basis set. In general, the largest momentum transferred in a Coulomb integral is 2*k*_cut_ if the states are aligned in parallel (in the opposite direction). If *k*_cut_ is increased, then Coulomb integrals with larger momentum transfer naturally occur in the calculations. However, also new integrals with zero momentum transfer (υ_αβ_^βα^) arise.

### 2.2 CC Theory

In this work, we employ single-reference CC theory to approximate electronic correlation effects. In CC theory the wave function is given by
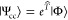
4where *T̂* is the cluster operator, and |Φ⟩ is the reference wave function. Throughout this work, we used a HF reference determinant. The full cluster operator for a system with *N* electrons can be written as
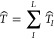
5which is typically truncated at some excitation level *L*.

#### 2.2.1 CC Doubles Theory

Since all single excitations are zero^42^ in the UEG, the lowest non-zero order CC ansatz is CCD, only including double excitations. The corresponding amplitudes *t*_*ij*_^*ab*^, which enter the cluster operator *T̂* in eq 4, are obtained from the following spin-free amplitude equation
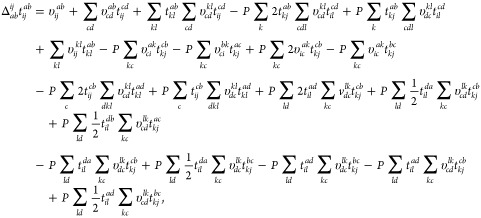
6with
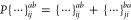
7The Δ_*ab*_^*ij*^ (and later Δ_*abc*_^*ijk*^) terms contain the HF eigenenergies and are defined by
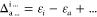
8The amplitude equation is solved iteratively until a self-consistent solution for the amplitudes *t*_*ij*_^*ab*^ is found. Note that it follows from momentum conservation of the Coulomb integrals (see eq 3), that all amplitudes *t*_*ij*_^*ab*^ not conserving momentum are zero. The converged amplitudes can then be used to evaluate the energy contribution beyond the HF energy, the so-called correlation energy
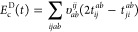
9We want to point out the well-known connection between second-order Møller–Plesset perturbation theory (MP2), third-order Møller–Plesset perturbation theory (MP3), and CCSD. Using only the first term on the right-hand side of eq 6, one retrieves the MP2 amplitudes, denoted by
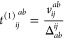
10Evaluating eq 9 with these amplitudes one obtains the MP2 correlation energy. We stress that for the UEG each element of the MP2 amplitude is completely described by the HF eigenenergies and the Coulomb integral. Thus, the elements of  will not change as the basis set size is increased. The equation for the MP3 amplitudes *t*^(2)^ can also be inferred directly from eq 6 by substituting *t*^(2)^ for *t* on the left-hand side and *t*^(1)^ for *t* for all contributions on the right-hand side that are linear in *t*, while disregarding the terms that are quadratic. Evaluating eq 9 with *t*^(2)^ yields the MP3 correlation energy. Note that the MP2 and MP3 correlation energies per electron diverge in the thermodynamic limit *N* → ∞.^11^ However, in the present case, we employ a simulation cell with a finite number of electrons where finite order perturbation theories also yield finite correlation energies.

#### 2.2.2 Triple Particle-Hole Excitations

The natural extension of CCSD would be the full inclusion of triple particle-hole excitation operators, denoted as CCSDT. This requires the solution of the corresponding amplitude equations for *t*_*ijk*_^*abc*^. However, the storage requirements of these additional terms is *N*_o_^3^*N*_v_^3^, which makes the approach impractical for larger system sizes. Hence, approximate CCSDT models have been investigated early on.^43−46^ In today’s calculations, the most popular among these methods is the CCSD(T)^47^ approach. Recently, a modified variant, known as the CCSD(cT) method,^12^ has been proposed. This approximation includes additional terms beyond the CCSD(T) method, providing a nondiverging description of zero-gap materials in the thermodynamic limit. As single excitations are absent in the UEG system, we present the CCD(T) and CCD(cT) methods. The correlation energy beyond *E*_c_^D^ for these methods is given by
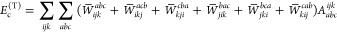
11with
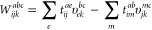
12where we define for any six-index quantity *x*_*ijk*_^*abc*^
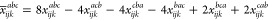
13The quantity *A* for the (T) model is given by
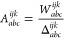
14with *W*_*abc*_^*ijk*^ = (*W*_*ijk*_^*abc*^)*.

In (cT), *A*_*abc*_^*ijk*^ contains further terms beyond those defined in eq 12. The full set of equations for this method is given in ref (12). Here, we provide the terms excluding all singles contributions. Instead of *W*_*ijk*_^*abc*^ as defined in eq 12, *W*_*ijk*_^′*abc*^ is used to construct *A*_*abc*_^*ijk*^, which is defined as
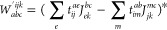
15with
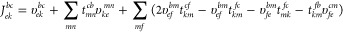
16
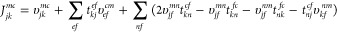
17

We emphasize that the large benefit of both approaches is the inclusion of triply excited clusters without storing an intermediate quantity of the size *N*_v_^3^*N*_o_^3^. Nevertheless, the memory footprint of (cT) is roughly doubled compared to that of the (T) approach. This is because *J*_*ek*_^*bc*^ is typically computed once and stored in memory. The computational cost for the evaluation of *J*_*ek*_^*bc*^ and *J*_*jk*_^*mc*^ is negligible compared to the contractions in eqs 12 and 15. Still, the total number of operations in (cT) is approximately twice that of the operations in a (T) calculation. In (T), we need to evaluate eq 12, which is the rate determining contraction scaling as . For (cT), however, one has to evaluate both the contractions in eqs 12 and 15.

## 3 Computational Details

All UEG MP2, CCD, CCD(T), and CCD(cT) calculations have been performed using a recently developed code.^48^ This code fully employs momentum conservation of the Coulomb integrals and amplitudes, resulting in a reduction of the storage requirements for CCD calculations from *N*^4^ to *N*^3^. Additionally, the number of operations in the CCD equations decreases from *N*^6^ to *N*^4^. Fully converged CCD amplitudes are obtained by solving the amplitudes equation eq 6 iteratively. We found an energy criterion of 10^–8^ a.u. sufficient for the analysis performed here. The memory requirements for CCSDT reduce from  to  when employing momentum conservation. However, in the UEG both (T) and (cT) can be implemented such that their memory requirements are , as it is for CCD. Accordingly, by employing momentum conservation the number of operations decreases from *N*^7^ to *N*^5^.

We stress that the presented results have only a weak dependence on the number of electrons in the unit cell. A larger electron number reduces the so-called finite-size error with respect to the thermodynamic limit. This error does not strongly interfere with the investigated BSIE. Importantly, the power-laws of the BSIE discussed here are fundamental and independent of the electron number. For the CCD analysis, we will work with a density of *r*_s_ = 5.0 a.u. with 54 electrons, while for the analysis of the triple excitations, with 14 electrons. The influence of the electron number and density will be further analyzed in Sections 4.3 and 5.3.

## 4 Large Momentum Limit Results for Various CCD Theories

We now turn to a discussion of the obtained results at the level of (approximate) CCD theory.

The BSIE originates from truncating the number of virtual states. In the UEG system, virtual states with high kinetic energy carry momentum that is large compared to the Fermi sphere radius defining the set of occupied orbitals. This implies that for the two virtual indices in eq 10, ***q*** ≈ ***k***_α_ ≈ – ***k***_β_. In this limit, the denominator of eq 10 is dominated by the kinetic energy contribution of the virtual states, and Δ_αβ_^*ij*^ ∝ *q*^2^, where *q* always represents |***q***|. The Coulomb integral υ_*ij*_^αβ^ becomes proportional to *q*^–2^, leading to an asymptotic behavior of . It is straightforward to assign a transfer momentum ***q*** to a Coulomb integral υ_*ij*_^αβ^ [see eq 3] and therewith to an amplitude *t*_*ij*_^αβ^.

When we increase the number of virtual states, i.e., enlarge the radius *k*_cut_, the number of accessible transfer vectors ***q*** increases accordingly. This implies for the energy expression in eq 9, , which is in accordance with the well-known *N*_v_^–1^ convergence behavior of the correlation energy (see ref (49)).

The BSIE Δ*E* is defined as the difference between the energy obtained from a calculation with a finite virtual basis set and the estimate from the CBS. In this work, CBS estimates of all channels are obtained by extrapolating energies from the two largest basis sets used for the given system, employing the corresponding power law.

### 4.1 Diagrammatic Contributions to the CCD Energy

In the present work, we partition the correlation energy and related quantities according to the right-hand-side contributions in eq 6. We refer to each contribution as a *channel*, aiming to identify distinct large-momentum behaviors in different channels, in order to improve or justify correction schemes for the BSIE. To this end, we introduce *channel amplitudes*, denoted as *t*^(*X*)^ and defined by the expression
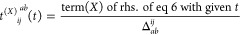
18where *X* represents one of the terms on the right-hand side of eq 6. For example, the MP2 and the PPL channel amplitudes stem from the first and the second term on the right-hand side of eq 6, denoted by (a) and (b), respectively. They can be expressed as  and . The channel amplitudes depend on the choice of approximation for the doubles amplitudes *t* on the right-hand side of eq 6. We examine three cases: (i) *t* = 0, (ii) *t* = *t*^(1)^, and (iii) the *t* that is the fully self-consistent solution of eq 6. In case (i), only the MP2 channel (a) is non-zero, yielding the MP2 amplitudes *t*^(a)^(0) = *t*^(1)^. In cases (ii) and (iii), all channels *t*^(*X*)^(*t*) are non-zero and depend on the argument amplitudes *t*. The first channel (a) always gives the MP2 amplitudes, as it is independent of *t*. We label the results obtained for cases (i), (ii), and (iii) as MP2, CCD^(1)^, and CCD, respectively. While a similar analysis was performed in a previous work for some contributions,^31^ our current study extends beyond the prior work. We also note that the contributions to CCD^(1)^ that are linear in *t*^(1)^ are identical to MP3.

**Figure 2:**
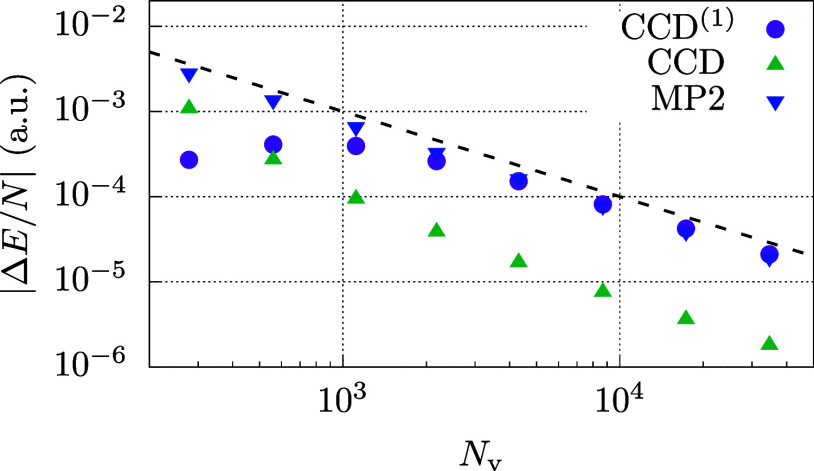
Plot displays the BSIE |Δ*E*| per electron for MP2, CCD^(1)^, and CCD. The dashed line is proportional to *N*_v_^–1^. Results are shown for the 54 electron system at a density *r*_s_ = 5.0 a.u. CBS estimates are obtained by an *N*_v_^–1^ extrapolation using the two largest systems.

Results for the density corresponding to *r*_s_ = 5.0 a.u. are presented in Figure 2. In all calculations, the BSIE converges like *N*_v_^–1^. However, we notice that the magnitude of Δ*E* for a given basis set differs significantly between MP2, CCD^(1)^, and CCD. Moreover, we note that Δ*E* approaches zero in the CBS limit with an opposite sign in CCD^(1)^ compared to MP2 and CCD. Consequently, the following question arises: *which contributions are responsible for these differences?*

**Figure 3:**
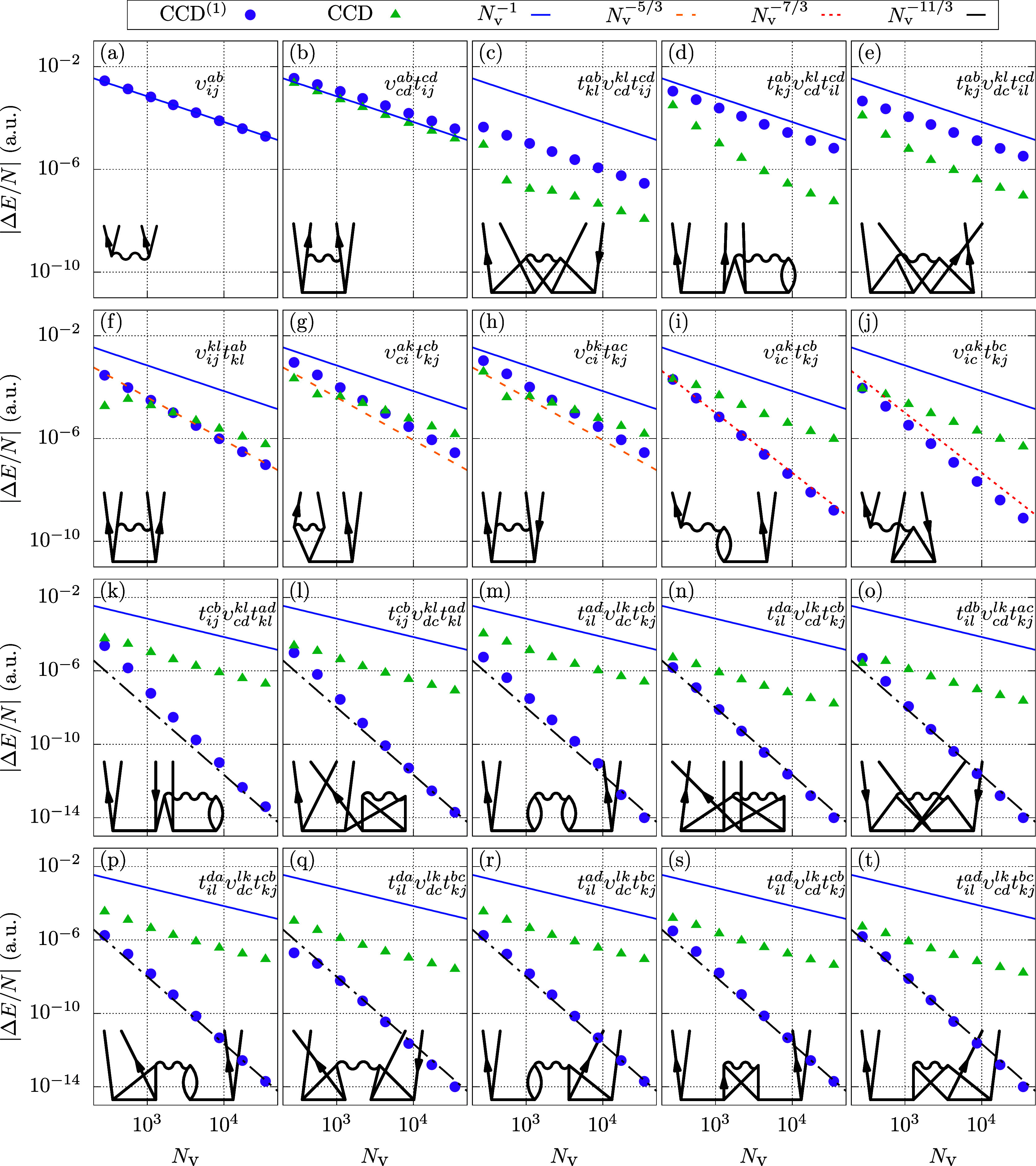
Shown are the absolute BSIEs |Δ*E*| per electron of all contributions to the CCD and CCD^(1)^ correlation energy expressions. The individual terms are given in order of appearance in eq 6. In order to simplify the identification, we provide the corresponding equations (where prefactors and sums are suppressed), as well as their diagrammatic representation. The lines of various color, indicating the different power laws, are identical in all plots. Results are shown for a system with 54 electrons and *r*_s_ = 5.0 a.u. CBS estimates are obtained from extrapolation of the two largest systems using the corresponding power law.

We now turn to a detailed analysis of the BSIE and the rate of convergence for all diagrammatic channels *t*^(*X*)^(*t*). These channels are computed from the given amplitudes *t*, which are either the MP2 amplitudes *t*^(1)^ or the solution of the amplitude equation [eq 6], referred to as CCD^(1)^ and CCD, respectively. Due to the large number of terms, this is a complex and elaborate endeavor. However, using a numerical approach, one can readily obtain all BSIEs using CCD^(1)^ and CCD theory. In Figure 3, we illustrate all individual BSIEs through 20 plots labeled (a–t). The organization of these plots is as follows: Figure 3a–e depict the BSIEs of all terms exhibiting a convergence of *N*_v_^–1^ at the level of CCD^(1)^ theory. Since MP2 is contained in both CCD^(1)^ and CCD, Figure 3a is identical to MP2 from Figure 2. For Figure 3f–h,i–j,k−t the CCD^(1)^ BSIEs exhibit convergence rates of *N*_v_^–5/3^, *N*_v_^–7/3^, and *N*_v_^–11/3^, respectively. In contrast, all CCD BSIEs converge as *N*_v_^–1^. The results depicted in Figure 3 show that the PPL term in Figure 3b is by far the most important contribution, besides the MP2 term, shown in Figure 3a. Consequently, our analysis begins with the PPL contribution. Signs and prefactors will be suppressed in the following analysis since we are only interested in the fundamental power laws.

#### 4.1.1 Particle–Particle Ladder Contribution

We now discuss the basis set convergence of the PPL contribution to the CCD^(1)^ and CCD energy. As depicted in Figure 3b, this contribution converges in both approaches at the same rate as the MP2 term, scaling as *N*_v_^–1^. The magnitude of the PPL contribution is found to be comparable to that of MP2, and especially in the case of CCD^(1)^, the PPL contribution is even larger than the MP2 term. These results have been obtained for the same density and number of electrons as in Figure 2. We have already discussed the significance of this contribution to the BSIE of CCSD theory in refs (30, 31, and 50), where different approaches have been presented to account for the BSIE of the PPL contribution. In refs (31 and 50), we also provide explanations for its *N*_v_^–1^ convergence rate. Here, we briefly reiterate the explanations by splitting the contributions to the amplitudes into conventional and augmented virtual basis sets. We suppress all contributions which contain amplitudes with one orbital in the finite- and the other in the augmented virtual basis set as these contributions are negligible [see Figure 1].
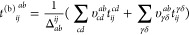
19
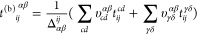
20The first term in the parentheses of eq 19 is the PPL contribution using the conventional finite basis. The second term in eq 19 reveals how amplitude elements from the augmented virtual basis couple to the amplitudes in the finite virtual basis set. In the UEG, we can approximate the appearing Coulomb interaction υ_γδ_^*ab*^ in the following way
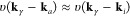
21This is well justified for high-lying augmented virtual states with very large momentum |***k***_γ_| ≫ |***k***_*a*_| > |***k***_*i*_|. Using this approximation, the last term in eq 19 can be written as . As discussed earlier, this term exhibits a 1/*N*_v_ convergence, indicating that the energy contribution derived from these amplitudes follows the same 1/*N*_v_ convergence behavior. Moving on to the first term in eq 20, we can employ the approximation introduced in eq 21. This allows us to write the term as . Carrying out the sum in the parentheses will lead to a scalar number for each electron pair *ij*. This number is related to the pair-specific correlation hole depth, introduced in previous work.^30^ We stress that this scalar number is dependent on the employed basis set.

**Figure 4:**
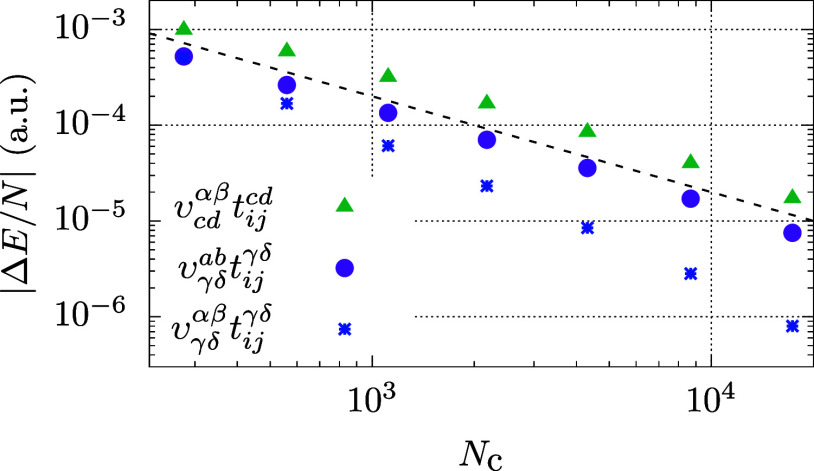
Energy contributions from three different terms given in eqs 19 and 20 using different cutoff *N*_c_ as discribed in the text. Results are obtained from fully converged CCD amplitudes using 54 electrons, *r*_s_ = 5.0 a.u., and 67,664 virtual orbitals. The dashed line is proportional to *N*_c_^–1^.

A central topic of discussion in the present work is the coupling of long- and short wavelength components of the amplitudes by the PPL term. *t*_*ij*_^αβ^ and *t*_*ij*_^*ab*^ can be considered short and long wavelength components, respectively. Terms that couple *t*_*ij*_^αβ^ to *t*_*ij*_^*ab*^ and vice versa can play an important role in the basis set convergence of all other diagrammatic contributions for increasing numbers of virtual orbitals. To better understand this coupling mechanism present, we investigate the basis set incompleteness error in  numerically. To this end, we take a short detour and analyze the three terms in eqs 19 and 20 that contain contributions from the augmented virtual basis set. We have computed converged CCD amplitudes using a very large virtual basis set with 67,664 virtual orbitals. Furthermore, the virtual states are partitioned into a *conventional* and an augmented virtual basis set, using *N*_c_ orbitals in the conventional basis set. With this partitioning, amplitudes and energies are evaluated using eqs 19, 20, and 9. Results for different numbers of *N*_c_ are shown in Figure 4. We stress that two contributions (∝υ_γδ_^*ab*^*t*_*ij*_^γδ^ and ∝υ_*cd*_^αβ^*t*_*ij*_^*cd*^) converge as *N*_v_^–1^, whereas the term containing υ_γδ_^αβ^ shows a faster convergence. The υ_*cd*_^αβ^*t*_*ij*_^*cd*^ term in eq 20 shows the largest BSIE in this calculation. However, we stress that due to the symmetry of the particle–particle term, the energy contribution from the second term in eq 19 is identical to the first term in eq 20 at the MP3 level of theory. On the one hand, this verifies numerically our theoretical analysis that the BSIE of the second term in eq 19 converges as slowly as *N*_v_^–1^. On the other hand this also demonstrates that the amplitude elements of the finite virtual basis set are altered significantly due the coupling of contributions from the (short wavelength limit) augmented virtual basis set. This alteration propagates to the other diagrammatic contributions even if they depend only on long wavelength amplitudes (*t*_*ij*_^*ab*^), and otherwise converge rapidly with *N*_v_.

#### 4.1.2 Slowly Converging Quadratic Contributions

We now seek to analyze the three contributions shown in Figure 3c–e. They are all quadratic in the amplitude *t*. Interestingly, these are the only contributions, beyond MP2 and PPL, showing an *N*_v_^–1^ convergence of the BSIE at CCD^(1)^ level of theory.

To explain this observation, we consider a two electron singlet system and split the contribution to the amplitudes into the conventional and augmented virtual basis sets. The three considered terms here are the third, fourth, and fifth terms on the right-hand side of eq 6. For the two electron system, these terms become identical, apart from different prefactors and read
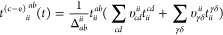
22
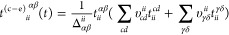
23The right term in the parentheses of eq 22 shows how the presence of the augmented basis alters the amplitudes belonging to the conventional basis set. The respective amplitudes are scaled by the terms in parentheses. For the two electron singlet, carrying out the sums over the two virtual states leads to the BSIE of the correlation energy of the electron pair, which converges slowly as *N*_v_^–1^. Thus, the quadratic contributions (c–e) exhibit the same power-law in both CCD^(1)^ and CCD calculations. The amplitudes from the augmented basis set of eq 23 show faster convergence. As both orbitals ϕ_α_ and ϕ_β_ are virtual states with large momenta, it follows that Δ_αβ_^ii^ ∝ *q*^2^ and *t*_*ii*_^αβ^ ∝ *q*^–4^. This results in an overall *q*^–6^ convergence of the corresponding amplitudes. Applying these amplitudes with augmented virtual states in the energy expression eq 9 leads to a *N*_v_^–5/3^ behavior of the BSIE.

Now we are in the position to explain the significant difference in magnitude of the BSIE for these contributions in CCD^(1)^ and CCD [see Figure 3c–e]. In the previous section, we have seen that the amplitude elements of the augmented virtual manifold are altered by the PPL contribution [eq 20]. For the studied system with 54 electrons at a density of *r*_s_ = 5.0 a.u., the amplitude elements from the augmented virtual states are found to be significantly smaller in CCD, than they are in CCD^(1)^. The predominant BSIE contribution stems from eq 22 which contains a contraction of such amplitude elements from augmented virtual states. This explains why the BSIE of the terms discussed here is larger in CCD^(1)^ compared to CCD.

We stress that this analysis, restricted to a two electron singlet, is evidently limited. The results for the 54 electron system in Figure 3c–e reveal differences of more than one order of magnitude between the different terms. However, for the two electron singlet, all terms are identical, apart from a factor of 2.

#### 4.1.3 Other Ladder Diagrams

This section addresses the three other ladder terms, linear in the amplitude *t*, shown in Figure 3f–h. For all three terms, the BSIE converges as *N*_v_^–5/3^ in the CCD^(1)^ calculations. We pick one of the terms, specifically a particle–hole term  depicted in Figure 3g, to study the origin of this convergence behavior. The remaining two terms can be treated analogously. We list all contributions after partitioning the virtual states into conventional and augmented virtual basis sets
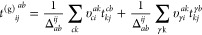
24
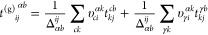
25
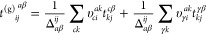
26
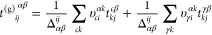
27The first term on the right-hand side of eq 24 is the conventional expression in the finite basis set. The second term in eq 24, as well as the terms in eqs 25 and 26 contain amplitudes or Coulomb integrals with one virtual orbital in the finite basis and the other in the augmented basis set. The vast majority of these terms are zero due to momentum conservation [see Figure 1]. Non-zero contributions can only be found in a volume corresponding to the Fermi sphere. As a consequence, these contributions are expected to be negligible compared to the terms in eq 27. Here, the first term again has one virtual state from the finite basis and the other from the augmented virtual states. Again, this contribution is negligible. The second term in eq 27 converges as *q*^–6^. This originates from the energy denominator 1/Δ_αβ_^*ij*^ and the amplitudes *t*_*kj*_^γβ^ scaling as *q*^–2^ and *q*^–4^, respectively. The maximum momentum transfer in the appearing Coulomb interaction cannot exceed 2*k*_F_. Thus, the Coulomb interaction does not introduce a further factor of *q*^–2^. Additionally, the sum over states *k* and γ does not alter the asymptotic behavior, as the number of states fulfilling the momentum conservation in the Coulomb integral is proportional to *k*_F_. In conclusion, the three ladder terms discussed here converge as *q*^–6^, corresponding to *N*_v_^–5/3^ in the energy.

Importantly, the BSIE behavior of ladder terms (f–h) changes fundamentally from *N*_v_^–5/3^ in CCD^(1)^ to *N*_v_^–1^ in CCD calculations. The origin of this effect lies in the PPL contribution and, to a smaller extent, in the other three quadratic contributions discussed in the previous section. Expressions such as eqs 19 and 22 couple the amplitudes of the augmented virtual states to the amplitudes of the conventional virtual states as discussed before. Since *t*_*ij*_^*ab*^ elements of the finite basis set on the right-hand side of eq 24 are considerably altered in a calculation with a larger basis set, the corresponding BSIE contribution of these channels is much larger. This means that the BSIE of these terms is not (directly) dominated by contributions in eqs 24–27 that contain augmented virtual basis functions.

#### 4.1.4 Linear Ring Type Diagrams

The terms depicted in Figure 3i–j are commonly referred to as ring and crossed ring diagrams. Similar to the previously discussed ladder diagrams, the ring and crossed ring diagrams also exhibit an *N*_v_^–1^ behavior in CCD, arising from the same underlying mechanism. In CCD^(1)^, however, the BSIE converges as *N*_v_^–7/3^. We present the ring contribution as an example (Figure 3i)
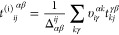
28which, in the limit of high-lying virtual states, exhibits *q*^–2^, *q*^–2^, and *q*^–4^ contributions, from 1/Δ_αβ_^*ij*^, υ_*i*γ_^α*k*^, and *t*_*kj*_^γβ^, respectively. Similar to the previously discussed particle−hole terms, the number of states *k* and γ which fulfill momentum conservation is proportional to *k*_F_. This results in an overall *q*^–8^ convergence of the amplitudes. In passing, we note that both diagrams in Figure 3i–j become identical in the large *q* regime, except for a different prefactor, while it is well-known that they behave very differently in the small *q* limit.^49^

#### 4.1.5 Other Quadratic Contributions

The remaining ten contributions in Figure 3k–t are all quadratic in the amplitudes *t*. The BSIE of these contributions shows a fast decay in CCD^(1)^, scaling as *N*_v_^–11/3^. As before, this behavior can be explained by the aggregation of factors *q*^–4^ for each amplitude, the factor *q*^–2^ once for the Coulomb interaction mediating the momentum *q* and once for the energy denominator 1/Δ_αβ_^*ij*^. Also here, the convergence behavior of the BSIE changes in the CCD calculation and becomes inversely proportional to the number of employed states, *N*_v_.

### 4.2 Structure Factor Analysis

The static structure factor is a pivotal quantity in the context of periodic electronic structure theory.^51^ In CC approaches, a related quantity, the so-called transition structure factor *S*, was employed in a number of recent works.^52−54^ The transition structure factor is directly related to the correlation energy contribution at a given momentum transfer ***q***, according to the following equation
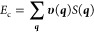
29For energy expressions in the form of eq 9, the transition structure factor can be written as
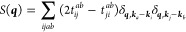
30where the amplitudes *t*_*ij*_^*ab*^ have been obtained from calculations with a given finite virtual basis set.

**Figure 5:**
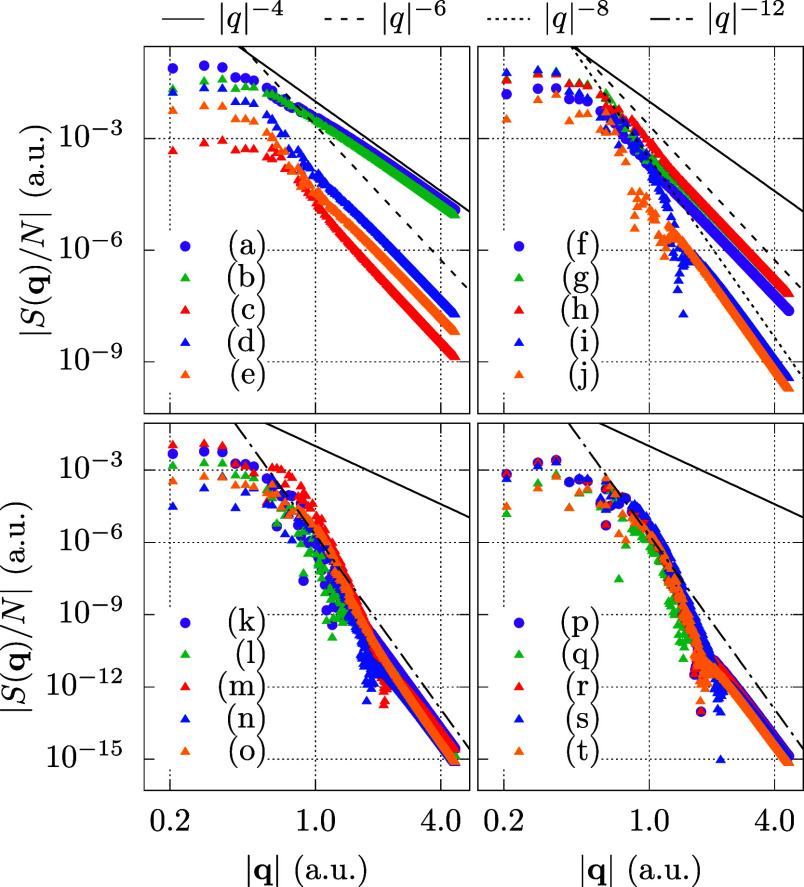
Transition structure factor results for the individual diagrammatic channels. Results are obtained from a converged CCD calculation with 54 electrons at *r*_s_ = 5.0 a.u., and 67,664 virtual orbitals. All figures show various diagrammatic contributions, labeled as in Figure 3. In addition, four lines with different powers of *q* are shown. The *q*^–4^ curve is the same in all plots and allows comparison between the four different panels.

As we have elaborated in the previous sections, in CCD the elements of the amplitudes depend on the employed basis set. Likewise, this holds for the transition structure factor, as indicated by eq 30. Consequently, we analyze the transition structure factor using a large basis set with >2500 virtual states per occupied orbital. We do not analyze results from CCD^(1)^ calculations but restrict to results from fully converged CCD amplitudes. The channel-resolved transition structure factor *S*^(*X*)^ can be defined by employing the respective channel amplitudes *t*^(*X*)^ in eq 30. Figure 5 displays the transition structure factors in the limit of large transfer momenta ***q***. This allows us to draw conclusions about the features at very short interelectronic distances. Interestingly, we identify only two channels that are of leading order for large ***q***, which is a central finding of the present work. These contributions correspond to the MP2 and PPL contributions, both showing a *q*^–4^ decay for large values of ***q***. It follows from eq 29 that this *q*^–4^ behavior of the transition structure factor corresponds to an *N*_v_^–1^ convergence of the BSIE, viz. . All other channels do not significantly alter the transition structure factor at large momentum transfers ***q***. Consequently, they cannot change the linear slope of the singlet transition pair correlation function at the coalescence point. We emphasize that the transition pair correlation function is not a ground state observable. However, these results corroborate that, for all channels besides MP2 and PPL, the slow *N*_v_^–1^ convergence of the BSIE actually arises from long-wavelength modulations of the corresponding channel decomposed transition structure.

### 4.3 Dependence on Electron Number and Density

In the previous sections, we carefully studied the BSIE of different contributions to the CCD correlation energy. We conducted the investigations for a single system of 54 electrons at the density *r*_s_ = 5.0 a.u. We saw that other than the MP2 term, the PPL contribution has by far the largest BSIE. As the BSIE of PPL and MP2 have opposite signs, this can lead to a significant decrease in the overall BSIE in CCD.

Here, we show how these main findings hold for different electron numbers and densities, respectively. However, we are not analyzing CCD^(1)^ but restrict the analysis to the CCD level of theory, which is the main interest of the present work. We analyze the BSIE of the different diagrammatic channels, using the fact that in CCD all channels show the same *N*_v_^−1^ decay. Table 1 shows results for three different electron numbers and two different electron densities, respectively. For the high density system, *r*_s_ = 1 a.u., all terms except the PPL terms show a very small contribution to the total BSIE. Only the PPL term has a significant BSIE compared to MP2, which is around 40%. The ratio of the PPL term is virtually independent of the system size for both densities considered.

**Table 1:** Shown is the Ratio of BSIEs between Different Channels and the MP2 term^a^

	*r*_s_ = 1.0			*r*_s_ = 5.0		
	*N*			*N*		
Δ*E*^(*X*)^/Δ*E*^(a)^	14	54	162	14	54	162
(b)	–410	–400	–404	–798	–795	–797
(c–e)	–11	–12	–8.3	0.9	–1.6	–3.7
(f–j)	–24	–15	–24	–97	–87	–80
(k–t)	–0.5	–2.5	–1.5	–1.2	–0.3	–0.9

a These ratios are obtained from calculations for different electrons *N* with consistent basis sets (*N*_v_/*N*_o_ ≈ 150–200). We have grouped the slowly quadratic contributions, i.e. (c–e), the linear ladder terms except PPL, (f–j), and finally all other quadratic terms (k–t). All ratios are scaled by 10^–3^.

In the low density system, at *r*_s_ = 5.0 a.u., all quadratic contributions, i.e., (c–e) and (k–t), show a small BSIE. The BSIE of the linear terms (f–j) is significantly larger compared to the high density system. The BSIE of the PPL contribution is around 80% of the MP2 value. Also the other linear terms in (f–j) are much more pronounced at low densities, with a BSIE approximately 10% of the BSIE from MP2. As for the other density, these results show no strong dependence on the employed electron number. We emphasize that the PPL contribution becomes more important in comparison to MP2 theory in the low density limit. This is expected because higher order perturbation theory terms become more significant as *r*_s_ increases. Furthermore, this implies that any truncated finite-order perturbation theory approach, such as CCD^(1)^, becomes unreliable. Indeed we find that CCD^(1)^ overestimates the PPL contribution compared with CCD significantly. However, similar to the resummation over ring diagrams, CCD performs a resummation over ladder diagrams, which is important for a well balanced estimate of the PPL contribution in the low density limit.

### 4.4 Discussion

Here, we performed an in-depth analysis of the BSIE of CCD for the UEG. We demonstrated that the MP2 and PPL contributions are the most important contributions to the overall BSIE. This is already well-known, especially in the context of explicitly correlated methods.^19,55^ Moreover, this fact was used in two different basis-set correction schemes developed by us in earlier works.^30,31^ In this work, we have illustrated the effectiveness of this ansatz. Table 1 shows that the PPL contribution leads to the major contribution of BSIE beyond MP2. This is valid for different electron numbers and densities. Furthermore, it is shown that especially the BSIE of the PPL contributions shows an opposite sign as the MP2 term in all studied systems. This implies that the overall BSIE of CCD is reduced when compared to an MP2 calculation. This effect becomes more prominent in the low density regime. However, we also stress that for systems with large *r*_s_, higher-orders of the cluster operator, beyond CCD, are required for an accurate description of the electronic system.^56^

**Figure 6:**
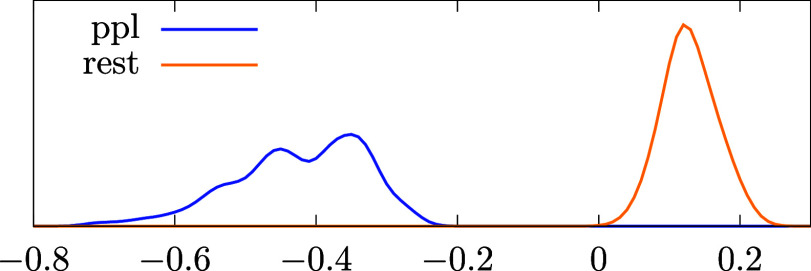
Ratio of BSIEs between different channels and the MP2 term Δ*E*^(*X*)^/Δ*E*^(a)^ for different molecular systems as described in the main text. ppl refers to Δ*E*^(b)^/Δ*E*^(a)^, whereas “rest” stands for Δ*E*^(c–t)^/Δ*E*^(a)^.

It is of great interest to what extent these findings are significant for (molecular) ab initio systems. Therefore, we analyze data from a molecular test set studied in a previous work^30^ which is freely available.^30^ For the 106 studied systems (the H atom is excluded), the BSIEs are analyzed similarly to Table 1. For these systems the AVTZ basis set is used as a finite basis set, whereas the CBS estimate is obtained from AV5Z/AV6Z extrapolation. Here, AVXZ stands for the aug-cc-pVXZ (or aug-cc-PV(X+d)Z for third row elements) basis sets.^57,58^ For the molecular results, the energy contributions are split into three channels, namely MP2, PPL, and all other terms (denoted as “rest”). Distributions of the BSIEs for these two channels can be found in Figure 6. This reaffirms that the largest contributions to the BSIE are in decreasing order given by MP2, PPL and the “rest”. A more detailed comparison of our findings for molecules depicted in Figure 6 and our results for the UEG summarized in Table 1 reveals, however, that the BSIE of the “rest” contribution exhibits a different sign than the BSIE of the PPL contribution for the molecules, whereas it has the same sign for the UEG.

## 5 Triple Excitations

This section is dedicated to the BSIE of the perturbative triples contributions presented in Section 2.2.2. We start the analysis with the (T) method and later discuss the differences for the (cT) method.

### 5.1 BSIE of the (T) Model

**Figure 7:**
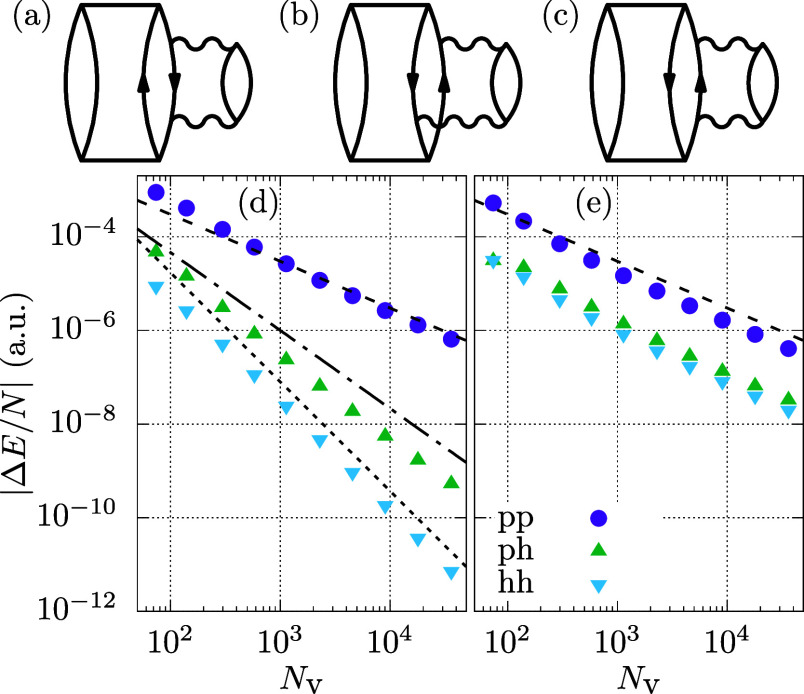
Figures (a–c) diagrammatically illustrate the three different contributions hh, ph, and pp, respectively. (d,e) display the BSIE per electron of the three mentioned contributions for a system with 14 electrons and *r*_s_ = 5 a.u. In (d), MP2 amplitudes are employed, while (e) shows results with CCD amplitudes. CBS estimates are obtained from extrapolation of the two largest systems using the corresponding power law. Three different lines in (d) show the different power laws, namely *N*_v_^–1^, *N*_v_^–5/3^, and *N*_v_^–7/3^.

The energy expression of the (T) contribution in eq 11 contains two different contractions, namely, a particle contraction and a hole contraction [eq 12]. As a consequence, the total energy expression can be split into three terms. One term which contains only hole contractions, denoted as (T)-hh in the following. A second term containing one hole and one particle contraction, (T)-ph. And finally, a term which contains two particle contractions, (T)-pp. The diagrammatic representations of these three terms is given in Figure 7a–c for nonpermuted terms. All three terms and permutations of the particles and holes add up to the full (T) energy.

We now analyze these three terms individually. In the following, we write the algebraic contributions without the intermediates defined in Section 2.2.2.

We start the analysis with the (T)-hh contribution, shown in Figure 7a, which reads for real-valued amplitudes without applying permutations
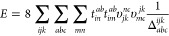
31We first examine the BSIE of this contribution using MP2 amplitudes. Figure 7d shows that the BSIE converges rapidly as *N*_v_^–7/3^. The expression contains the sum of three virtual states. The sum over the state *c* does not provide an energy contribution for sufficiently high-lying virtual states. This is because the appearing Coulomb integrals contain three states from the set of occupied orbitals. Consequently, all Coulomb integrals where the wave vector of the virtual state exceeds 3*k*_F_ are zero because of momentum conservation. Therefore, an energy contribution from such high-lying virtual states can only stem from the sums over the states *a* and *b*. As mentioned earlier, contributions of type *t*_*ij*_^α*b*^ have a negligibly small contribution. These non-negligible contributions, using the notation of augmented virtual states, can be written as
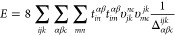
32It is straightforward to derive the fundamental convergence behavior of eq 32. In the limit of virtual states with high energy, the wave vectors of states inside the Fermi sphere become negligible and the amplitudes converge as *q*^–4^. The energy denominator 1/Δ_*ijk*_^αβ*c*^ converges as *q*^–2^. Thus, in the limit of large *q* the energy expression can be expressed as , which is in accordance with the observed *N*_v_^–7/3^ behavior of the BSIE. When applying the permutations in eqs 11 and 13 the BSIE of some contributions is zero for sufficiently large transfer momenta *q* as none of the additional contributions fulfill the corresponding momentum conservation.

We now consider the case where the CCD amplitudes are employed in the energy expression for *E*^(T)-hh^. The BSIE for this case is depicted in Figure 7e and exhibits a clear *N*_v_^–1^ behavior. The identical effect was observed in Sections 4.1.3, 4.1.4, and 4.1.5, and can be explained by the basis set incompleteness of the employed amplitudes *t*_*ij*_^*ab*^ within the finite basis set.

The (T)-ph contribution, depicted in Figure 7b, shows the same convergence behavior as (T)-hh only when amplitudes from the CCD are employed. However, as shown in Figure 7d, employing MP2 amplitudes, the BSIE converges with *N*^–5/3^. Interestingly, the term without permutations, as illustrated in Figure 7b, converges faster, namely, as *N*^–7/3^. Here, one of the Coulomb integrals contains three occupied states, which prevents any contributions from sufficiently high-lying states *c*. This implies that the momentum transfer of the other appearing Coulomb integral υ_*bc*_^*kf*^ is also restricted to the order of magnitude of *k*_F_. However, when employing the permutations in eqs 11 and 13 some of the contributions show a slower convergence rate, namely *N*^–5/3^. This can be seen exemplarily for the following contribution
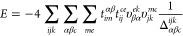
33This energy expression is one particular contribution of the full (T) energy, as given in eq 11. It is obtained by taking only contraction of the first element  in the parentheses of eq 11, where  does not contain all permutations from eq 13 but only the one where *a* and *c* are swapped. In eq 33 the virtual and augmented virtual states are chosen such that they lead to the dominant contribution for this term. In this expression one of the occurring set of amplitudes contains states from the augmented virtual states, whereas the other can incorporate states from the finite virtual basis set. Consequently, the expression is piling up the factors *q*^–4^, *q*^–2^, and *q*^–2^ from the amplitudes, Coulomb integral, and energy denominator, respectively. The final BSIE is found by the corresponding limit .

Finally, we discuss contribution (T)-pp, shown in Figure 7c, which shows a much larger BSIE than the two other discussed contributions, as can be seen from Figure 7d,e. The BSIE of this contribution is *N*_v_^–1^ for both MP2 and CCD amplitudes. Here both appearing Coulomb interactions contain three particle and one hole state. Now, amplitudes containing states from the finite virtual basis set can couple to augmented virtual states by the Coulomb interactions. The corresponding expression, written with the notation of the finite and augmented virtual states reads
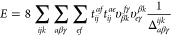
34This leads to a completely different behavior for large values of *q*. The two occurring amplitudes belong to the finite virtual basis set and do not introduce a further power of *q*^–4^, however, the Coulomb interactions now scale as *q*^–2^ resulting in a BSIE proportional to . These terms, as given in eq 34, dominate the BSIE of the (T)-pp term and, therefore, the BSIE of the whole (T) contribution. Contributions where the amplitudes contain states from the augmented virtual basis set show a convergence of *N*_v_^–5/3^ or faster. We note that some of the permutations in eqs 11 and 13 show faster converging contributions. We emphasize that these findings are not entirely novel. Empirically, it is well-known that the leading rate of convergence of (T) is similar to MP2.^59^ This even led to ad-hoc correction schemes that rescale the (T) contributions with MP2 terms to correct for the BSIE of the (T).^60,61^ Additionally, Köhn incorporated the explicitly correlated framework for the (T) contribution.^62^ He was able to numerically identify that the terms given in eq 34 cover the important contributions to the BSIE.^63^

### 5.2 (T) Vs (cT)

In this section, we analyze the BSIE of the recently proposed complete perturbative triples correction (cT). The motivation for this work was to incorporate additional contributions to prevent the perturbative correction from diverging for metallic systems^12^ in the thermodynamic limit. These further contributions are given in eqs 16 and 17. In total, ten further terms beyond the bare Coulomb interactions in eqs 16 and 17 are included. In passing, we note that only two of these additional terms are accountable for resolving the divergence for metallic systems in the (T) method, namely the two ring terms υ_*ef*_^*bm*^*t*_*km*_^*cf*^ and υ_*jf*_^*mn*^*t*_*kn*_^*cf*^. The analysis performed in the previous work focused on the small-*q* regime where the divergence occurs. In this work, we analyze the behavior for large *q*, i.e., studying the BSIE of the (cT) model.

**Figure 8:**
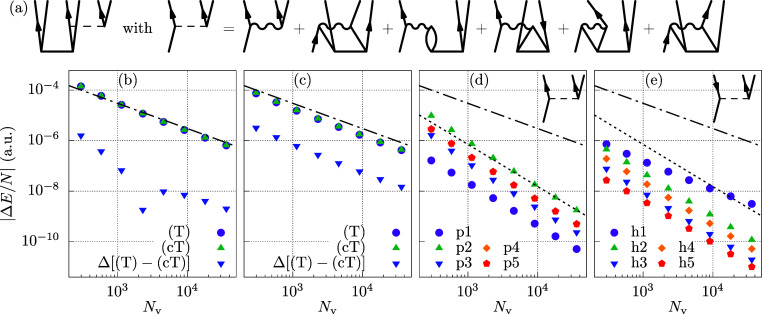
(a) Diagrammatic illustration of the terms in (cT) theory as given in eq 16. All terms connecting to another doubles amplitude on the right are additional terms of (cT), labeled 1–5 in order of their appearance. These terms are not occurring in the (T) theory. (b,c) Shown is the BSIE per electron of the (T) and (cT) contribution for a system with 14 electrons at a density *r*_s_ = 5.0 a.u. Further shown is the convergence of the energy difference between (T) and (cT). (b,c) show results for MP2 and CCD, respectively. (d,e): p1–5 are the five additional diagrams emerging from eq 16. h1–5 are the corresponding terms from eq 17. CBS estimates are obtained from extrapolation of the two largest systems using the corresponding power law. The dashed-dotted and the dotted lines are proportional to *N*_v_^–1^ and *N*_v_^–5/3^, respectively.

For these reasons, we analyzed the convergence of the energy expressions (T) and (cT) for the same system. Similar to the analysis in CCD, the BSIE is shown for MP2 amplitudes and converged CCD amplitudes, shown in Figure 8b and c, respectively. It can be seen that the BSIE of the additional terms in (cT) are at least one order of magnitude smaller than the BSIE of the (T) expression. The difference between (T) and (cT) is larger when using CCD amplitudes, however, this difference shows also a slow *N*_v_^–1^ convergence when using MP2 amplitudes. This analysis reveals that at least one of the additional terms is expected to cause a slow *N*_v_^–1^ convergence. Consequently, we perform an analysis of the ten additional contributions individually. The diagrammatic contributions are depicted in Figure 8a. We note that the first diagram on the right-hand side corresponds to (T), whereas all others are included in (cT) and are referred to as h/p1–h/p5. Again, we use amplitudes from MP2 level of theory for the following analysis as all terms show an *N*_v_^–1^ with CCD amplitudes.

The BSIEs of the individual terms are given in Figure 8d,e. Evidently, only one of these terms is slowly converging, whereas all other contributions converge as *N*_v_^–5/3^. Interestingly, the slowly converging h1 contribution is very similar to one of the slowly convergent terms in the CCD expression (viz., Figure 3d).

### 5.3 Dependence on Electron Number and Density

**Figure 9:**
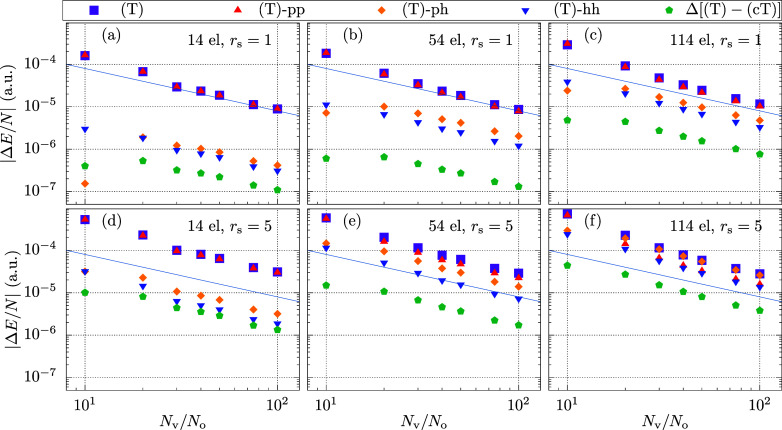
BSIE per electron for different contributions are shown. (a–c) show results for *r*_s_ = 1 a.u., whereas (d–f) uses a density of *r*_s_ = 5 a.u. Panels in the left, middle, and right column show different system sizes with 14, 54, and 114 electrons, respectively. To allow comparison between systems with different electron numbers we present the results in BSIE per electron, and the number of virtual states per occupied orbital. The blue line is proportional to *N*_v_^–1^ and allows comparison between the six different panels.

Up to here, the analysis of the BSIE of the triples contributions was done for a system with 14 electrons and a density of *r*_s_ = 5 a.u. In this section, we want to study two different densities, *r*_s_ = 1 a.u., and *r*_s_ = 5 a.u., as well as three different system sizes, namely 14, 54, and 114 electrons. Figure 9 summarizes the BSIE of the quantities discussed in the previous sections, viz. the contributions of the previously defined channels T-pp, T-ph, and T-hh, as well as the difference between the (T) and the (cT) method.

For all analyzed systems, the difference between (T) and (cT) is at least around one order of magnitude smaller than the BSIE of (T). It can be observed that the terms beyond (T) in (cT) are more important for larger electron numbers as well as for larger *r*_s_.

We move on to the discussion of the three channels (T)-pp, (T)-ph, and (T)-hh. For small values of *r*_s_, as well as for small electron numbers, (T)-pp dominates the BSIE of the overall BSIE of (T). However, the BSIE of the other two channels increases on a relative scale if the number of electrons or the value of *r*_s_ is increased, respectively. For the system with 114 electrons and a density of *r*_s_ = 5.0 a.u., the (T)-ph term is already the largest in magnitude. It is important to note that, as discussed in the previous section, the *N*_v_^–1^ decay of (T)-ph and (T)-hh does not stem from contributions with large momenta *q*. The BSIE rather originates from the changes in the CCD amplitude elements of the finite virtual basis set. These two leading order contributions, namely the basis set incompleteness in the CCD amplitudes and the additional contributions from *W*_*ijk*_^*a*βγ^ as given in eq 34 have been already identified by Köhn^62,63^ in the context of an F12 correction for the (T) contribution. The analysis performed here shows that the relative importance of one contribution or the other depends strongly on the studied system.

## 6 Summary and Conclusions

In this work, we present a detailed analysis of different coupled-cluster theories applied to the UEG in the large-momentum-transfer limit. This allows a fundamental investigation of the BSIE in these theories. Even though it is well-known that the MP2 and PPL contributions are the most dominant terms in CCD, this work investigates all further terms in CCD. In particular, we have shown that in MP3 theory, which corresponds to a low-order approximation of CCD (here referred to as the linear terms of CCD^(1)^), all diagrammatic contributions besides MP2 and PPL converge fundamentally faster, namely as *N*_v_^–5/3^ or *N*_v_^–7/3^. In CCD theory, however, all diagrammatic contributions converge slowly as *N*_v_^–1^. This is because the PPL term and other contributions couple amplitude elements with small and large momenta. Nevertheless, it has been shown for different densities and electron numbers that MP2 and PPL are throughout the dominant terms. This is also evident in the transition structure factor, which shows only for the MP2 and PPL terms a *q*^–4^ decay for large values of *q*. All other diagrammatic contributions show faster convergence behavior. It was further shown that these key findings are valid in the range of realistic densities, 1.0 ≤ *r*_s_ ≤ 5.0, and show only little dependence on the number of electrons in the system.

For the (T) contribution, a similar decomposition into three channels was carried out. It was shown that only one of the channels shows a leading order contribution when using MP2 amplitudes. In CCD, however, due to the basis set incompleteness of the employed doubles amplitudes, all three channels show the same *N*_v_^–1^ decay. The relative strength of the three different channels to the total BSIE depends on the number of electrons and the employed density. It was shown that there are essentially two major contributions to the overall BSIE: (i) a term that stems from elements with large momentum transfer on the additional Coulomb interaction of (T), which is only dominantly present in the so-called (T)-pp channel and (ii) contributions that are linked to the basis set incompleteness of the underlying CCD amplitudes. The latter contribution is significant for all three channels: (T)-pp, (T)-ph, and (T)-hh.

Finally, the BSIE of the recently proposed (cT) approach was investigated. It was shown that the additional contributions beyond the (T) approach show only a small BSIE. This is desirable as this new approach was conceived as a nondiverging perturbative triples correction for vanishing-gap systems. The mentioned divergence occurs for small momentum transfers, whereas the BSIE is attributed mainly to the regime of large momentum transfers.
